# Partial laryngectomy and reconstruction with rotation of the epiglottis in the treatment of a rare laryngeal schwannoma: a case report 

**DOI:** 10.1186/s13256-020-02537-z

**Published:** 2020-11-25

**Authors:** Claudiney Candido Costa, Hugo Valter Lisboa Ramos, Wilder Alves, Pauliana Lamounier, Leandro de Castro Velasco, Mikhael Romanholo El Cheikh

**Affiliations:** Department of Otorhinolaryngology and Head and Neck Surgery, CRER - Centro de Reabilitação e Readaptação Dr. Henrique Santillo, Goiânia, Goiás, Av. Ver. José Monteiro, 1655 - Negrão de Lima, Goiânia, 74653-230 Brazil

**Keywords:** Laryngeal schwannoma, Larynx, Neurogenic tumors

## Abstract

**Background:**

Neurogenic tumors of the larynx are rare, with few cases having been reported in the literature. Schwannomas are responsible for 0.1% of all benign tumors of the larynx. They arise in the sheaths of the peripheral, autonomic, and cranial nerves. The objective of this report is to describe a case of a bulky laryngeal schwannoma, the surgical procedure for its removal, and the long-term patient follow-up.

**Case presentation:**

A 19-year-old Brazilian woman presented to our institution with a complaint of progressive dyspnea over the preceding year, as well as dysphonia, dysphagia for solids, and globus pharyngeus. Direct rigid laryngoscopy showed a supraglottic tumor obstructing approximately 90% of the larynx. With the symptoms progressing to severe dyspnea, an emergency tracheostomy was performed. After infusion of intravenous contrast, magnetic resonance imaging revealed a mass lesion with intense, heterogeneous contrast enhancement along the posterior wall of the hypopharynx, blocking all of the lumen and measuring 2.8 cm and 2.2 cm at its largest diameters. The image suggested a neoplastic lesion. The patient underwent open surgery for tumor resection. Her postsurgical recovery was uncomplicated. Histopathology and immunohistochemistry revealed the tumor to be a laryngeal schwannoma.

**Conclusion:**

The definitive diagnosis of laryngeal lesions can be difficult, and histopathology plays a pivotal role. Laryngeal schwannomas are rare; however, tumors can become large and may ultimately lead to airway obstruction.

## Background

Schwannomas are rare peripheral nerve tumors that originate from Schwann cells in the motor, sensory, and cranial nerves [1]. Neurogenic tumors rarely involve the larynx, representing only 0.1–1.5% of all benign laryngeal tumors [1, 2]. There are two types of neurogenic tumors—schwannomas and neurofibromas—with 45% occurring in the head and neck region [3]. Laryngeal schwannomas account for approximately 0.1% of all benign tumors of the larynx. The most common site involved is the aryepiglottic fold, and the nerve from which it usually originates is the superior laryngeal nerve [4, 5]. There are few cases of this type of tumor reported in the literature. We describe a case of a patient with a bulky tumor treated satisfactorily by the external route.

## Case presentation

**Fig. 1:**
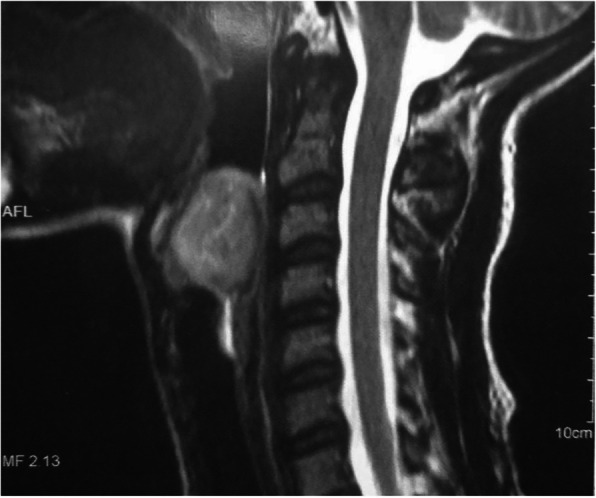
Magnetic resonance imaging (MRI) showing a well-circumscribed, clearly outlined supraglottic tumor with regular edges and hyperintensity in T2-weighted images, causing significant obstruction of the laryngeal lumen

A 19-year-old Brazilian woman who was a nonsmoker and a student was admitted to the emergency ear, nose, and throat department of a university hospital complaining of dysphonia and progressive dyspnea, mainly exercise-related, over the course of approximately 1 year. During videolaryngoscopy, a massive supraglottic lesion was found originating in the left aryepiglottic fold and causing significant obstruction of the laryngeal lumen. The patient required an emergency tracheostomy, and biopsy was performed by direct laryngoscopy. Magnetic resonance imaging (MRI) showed a well-circumscribed, clearly outlined tumor with regular edges and hyperintensity in T2-weighted images (Fig. 1).

Histopathology revealed a proliferation of spindle cells with indistinct cytoplasmic boundaries. The cells were arranged in compact bundles or interlacing fascicles, forming areas of nuclear palisading and Verocay bodies. Few typical mitotic figures were found. There were few hypocellular areas with loosely arranged myxoid matrix. Immunohistochemistry showed positivity for S100 protein, confirming the diagnosis of schwannoma.

**Fig. 2:**
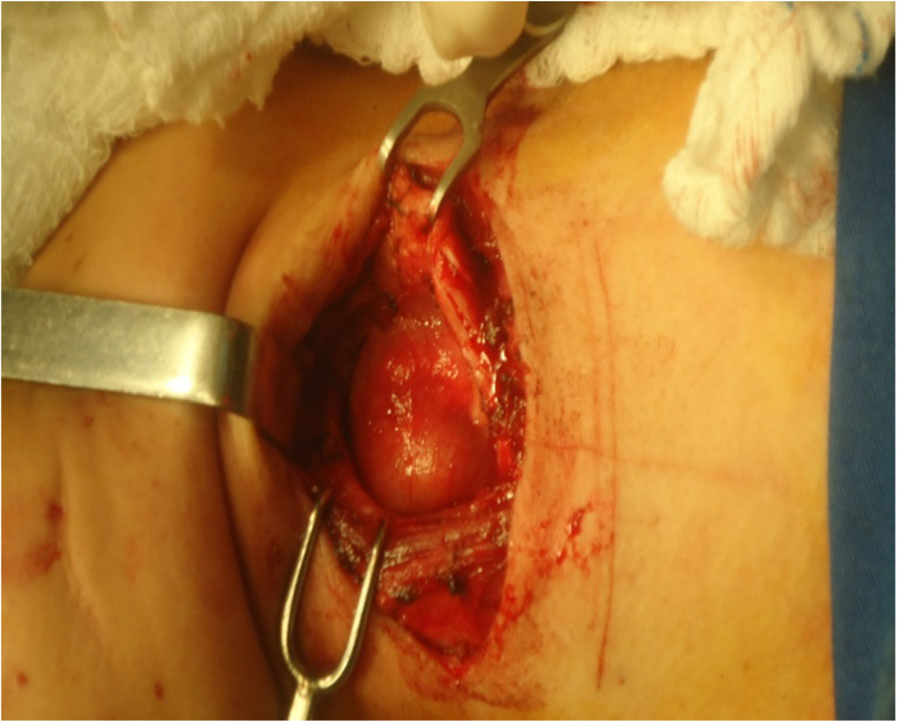
External surgery by laryngofissure (or median thyrotomy) in view of the size and location of the lesion. The lesion affected the aryepiglottic fold, arytenoids, and left vocal cord

Because of the size and location of the lesion, an external surgical procedure was performed, with the tumor being excised via a laryngofissure approach. The lesion involved the aryepiglottic fold, the arytenoid cartilages, and the left vocal cord (Fig. 2). Excision of the tumor therefore also required removal of the aryepiglottic fold, the ventricular band, and the upper two-thirds of the left arytenoid, preserving the vocal process of the left arytenoid cartilage. The petiole of the epiglottis was rotated to the left and reinserted to help supraglottic closure. Histopathological results confirmed the diagnosis of schwannoma, with the surgical margins being free of disease.

**Fig. 3:**
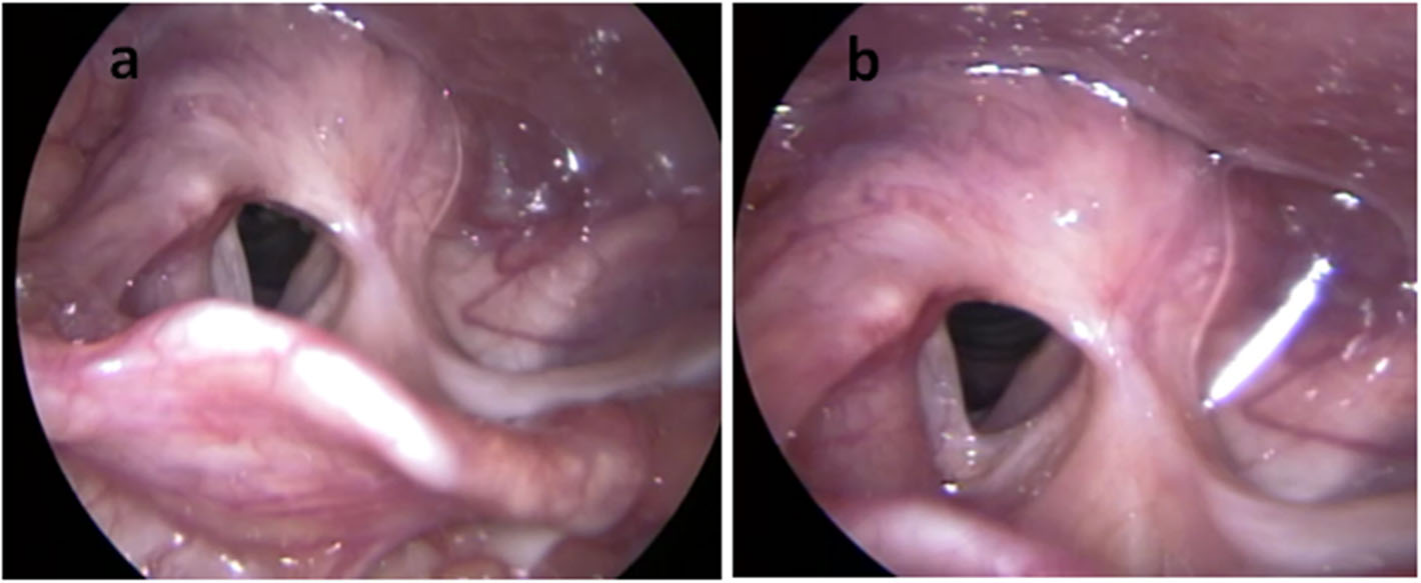
Direct videolaryngoscopic image obtained 3 years after surgery. **a** Rotation of the left epiglottis petiole. **b** Arytenoidectomy and partial left cordectomy leading to a satisfactory glottis lumen

**Fig. 4:**
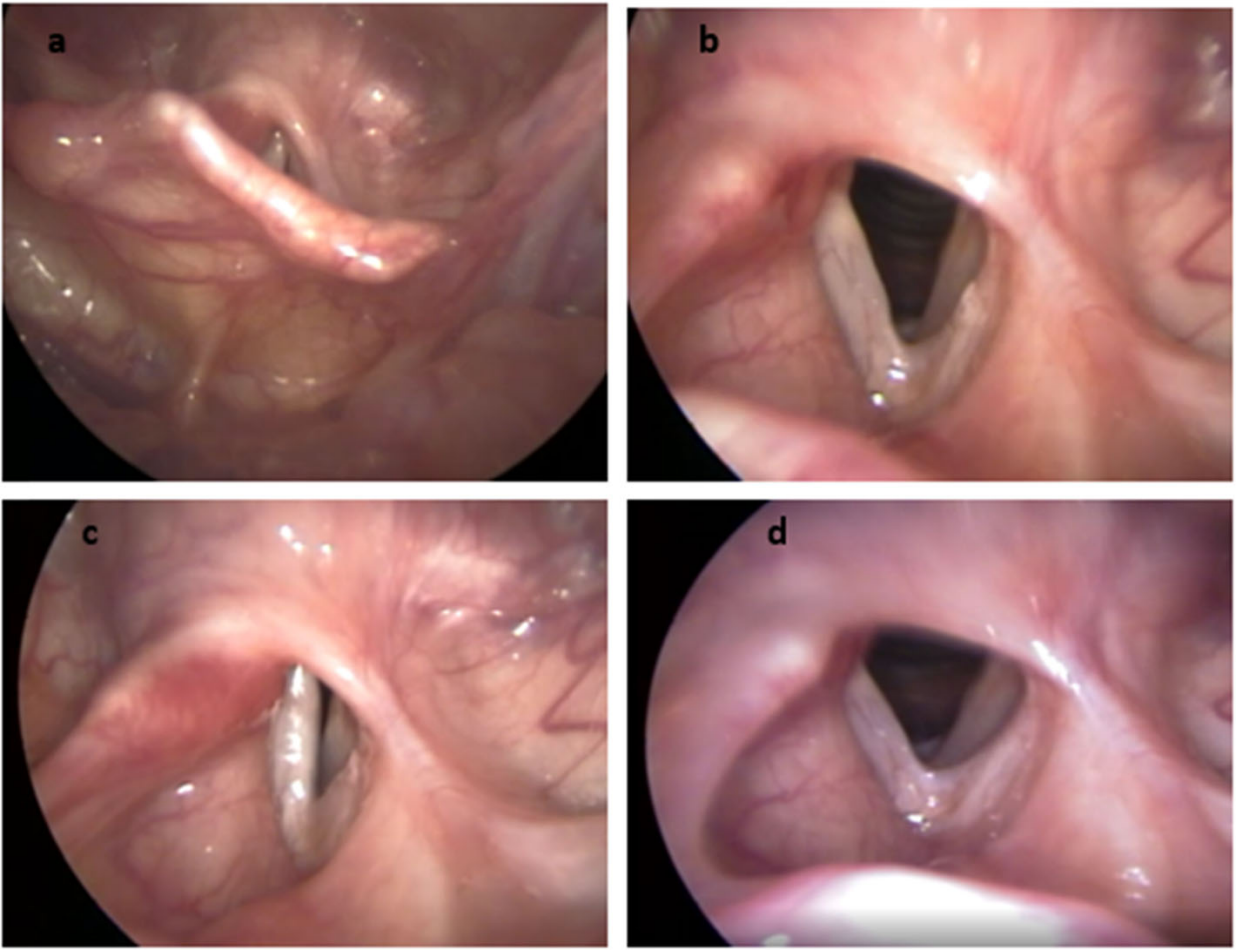
Direct videolaryngoscopic image obtained 8 years after surgery. **a** Rotation of the left epiglottis petiole for reconstruction of the glottis region. **b** Arytenoidectomy and partial left cordectomy leading to a satisfactory glottis lumen. **c** Arytenoidectomy and partial cordectomy on the left side, resulting in satisfactory lumen glottis. **d** Glottal closure with posterior triangular cleft compensated by vocal “neofold” (fibrosis? epiglottis petiole rotated?) on the left

The patient recovered satisfactorily, with the tracheostomy and nasogastric tubes being removed 15 days after surgery. The patient’s swallowing was normal, and her voice quality was good. The patient was seen in follow-up for 8 years with no signs of recurrence of the lesion (Figs. 3 and 4).

## Discussion and conclusion

Laryngeal schwannoma is a rare benign tumor and is most often located in the aryepiglottic fold [6–8]; nevertheless, in our patient’s case, the lesion was also found in the vocal fold and arytenoids. The symptoms of this disease depend on the location, size, and growth rate of the tumor; however, they include persistence of globus pharyngeus symptoms, dysphonia, and dyspnea, and some patients may have stridor [3–9]. There is one reported case of death due to asphyxia caused by a laryngeal schwannoma [10]. In the case reported here, the patient required an emergency tracheostomy because she presented at the medical institute with significant dyspnea resulting from the presence of the bulky tumor.

The MRI findings for the diagnosis of schwannoma usually consist of an isointense to slightly hyperintense lesion on T1 and possible hyperintensity on T2. The lesion is well-defined and of a benign appearance [11, 12]. In our patient’s case, MRI revealed a T2 hyperintense image of a bulky tumor with regular borders in the supraglottic region (Fig. 2).

Differential diagnoses include chondroma and adenoma, with a diagnosis of schwannoma being confirmed by histopathology, in which cellular and hypocellular areas alternate in varying proportions and are known as Antoni A and Antoni B cellular patterns. The Antoni A pattern is characterized by dense proliferation of cells, and its structural arrangement forms a palisade pattern. The Antoni B pattern is not characterized by any particular structural arrangement. Both types can be found in the same tumor. Immunohistochemistry shows reactivity for S100 protein.

The treatment of choice is complete excision of the lesion, which can be performed by endoscopy or external surgery. The size of the tumor, the site of the lesion, the damage to the structures of the larynx, the surgeon’s preference, and the risk of mucosal damage all play a role in the choice of the surgical technique. The size of the lesion is the principal determinant of the route of access [13, 14]. There is controversy in the literature with respect to preoperative tracheostomy. Our patient required the procedure due to airway obstruction. The external approach can be performed by lateral pharyngotomy or laryngofissure, which was the method used in our patient’s case due to the site of the tumor.

Schwannoma of the larynx is a rare tumor; however, it may grow to a considerable size and can even cause airway obstruction. The size and location are factors that determine the choice of the surgical technique, with complete resection and maximum preservation of laryngeal physiology being the principal goals.

## Data Availability

Not applicable.
